# Self-reported cognitive functioning on the behavior rating inventory of executive function in psychotic disorders: Effects of gender, education and cognitive functioning

**DOI:** 10.1016/j.scog.2025.100399

**Published:** 2025-10-28

**Authors:** Eivind Haga Ronold, Rune Raudeberg

**Affiliations:** a Department of Biological and Medical Psychology, University of Bergen, Postboks 7807 NO, 5020, Bergen, Norway; b Mohn Medical Imaging and Visualization Centre, Department of Radiology, Haukeland University Hospital, Post Office Box 1400 N, 5021, Bergen, Norway.

## Abstract

**Introduction:**

Psychotic disorders (PD) are among the most severe mental disorders and cognitive impairment contributes to this. Few studies have investigated self-reported executive function (srEF)in PD using the Behavior Rating Inventory of Executive Function Adult version (BRIEF-A) and have found varying results. Population characteristics could contribute to this heterogeneity. The current study thus reported BRIEF-A scores in a sample with PD, and how this was affected by gender, education and cognitive function measured by The Repeatable Battery for the Assessment of Neuropsychological Status (RBANS).

**Methods:**

BRIEF-A consist of 9 scales, two indices (Behavioral Regulation Index [BRI] and Metacognition Index [MI]), ands a summary score Global Executive Composite (GEC). T-scores (high score = difficulties) from 260 patients (99 women, mean age 24.58) was included. Independent samples t-tests were used to compare means between genders. Gender, RBANS Total Scale scores and years of education were used to predict BRIEF-A scores in general linear mixed models.

**Results:**

For the full sample, the BRIEF-A the GEC showed a marginally impaired T-score (T >60) and the MI showed highest scores. Females had a GEC and MI score above clinical cutoff (T ≥65), and had significantly more srEF difficulties on all scales except Inhibit and Self-Monitor. Gender and global cognition showed some multivariate interactions, but these effects were small.

**Conclusions:**

The current study indicates srEF marginally impaired meta-cognition in PD, enhanced by gender. Future studies should investigate srEF longitudinally to see how this develops following psychosis onset and identify groups in need of targeted treatments like cognitive remediation.

## 1 Introduction

Psychotic disorders (PD) are arguably the most severe mental conditions of all. The significant cognitive impairment experienced by many individuals with these disorders contributes to this (Harvey et al., 2019). Although many studies have investigated how people with PD show lower performance than both healthy control- and mood disorder populations on cognitive tests (Carvalho et al., 2015), relatively few have investigated subjectively reported cognitive difficulties, particularly for reported executive functioning (srEF). In a systematic review, Groenman et al. (2022) found that subjectively srEF showed small and inconsistent associations to objective tests in studies of patients with schizophrenia. In addition, srEF was related to global functioning. The included studies showed inconsistent associations, however, and only included one scale for srEF, specifically the Behavior Rating Inventory of Executive Function Adult version (BRIEF-A (Roth et al., 2005)). BRIEF-A could thus be a clinically relevant srEF measure for people with PD, but more studies are needed to establish its utility in this population.

Executive functioning is a central cognitive function important for planning, organizing, initiating, selecting and controlling responses in daily life (Snyder et al., 2015). Executive functions measured by cognitive tests are often broken down into inhibition, working memory/updating, and shifting, and are essential for solving other cognitive tasks like learning and memory, that show deficits across psychiatric disorders (Snyder et al., 2015). An umbrella review of meta-analyses of cognitive test results for people with schizophrenia found that executive function was the second most investigated cognitive domain, with findings showing impairments in PD with working memory being particularly effected (Gebreegziabhere et al., 2022). In contrast, fewer studies have investigated srEF in PD (Groenman et al., 2022). Increasingly, the importance of adding srEF measures into assessments of cognition in psychiatric populations are being recognized as providing ecologically valid data that can supplement results from objective cognitive tests (Friedman and Gustavson, 2022; Miskowiak et al., 2022; Fuermaier et al., 2015). In addition, the BRIEF-A measures aspects of executive functioning not captured by traditional cognitive tests, such as emotional control and initiation (Roth et al., 2005). Thus, knowing how established instruments like BRIEF-A captures the cognitive difficulties experienced in various clinical populations could be important for understanding the cognitive profile in PD.

BRIEF-A is a standardized and normed informant based and srEF questionnaire (Roth et al., 2005). BRIEF-A measures many of the central aspects of executive functions mentioned above, and has composites for Behavior Regulation (BRI: Consisting of Inhibit, Shift, Emotional Control, and Self-Monitor scales), Metacognition (MI: Consisting of Initiate, Working Memory, Plan/Organize, Task Monitor, and Organization of Materials scales), and a Global Executive Composite score (GEC: Consisting of all scales), in addition to three validity scales (Inconsistency, Negativity, and Infrequency).

BRIEF-A has been reported to correlate with several important outcomes in PD, including functioning and quality of life (Groenman et al., 2022; Glenthøj et al., 2020), personal recovery (Van Aken et al., 2022), social functioning (Van Aken et al., 2025), and symptoms and disability (Randers et al., 2020; Van Aken et al., 2023). In addition, BRIEF-A has been shown to capture improvements following cognitive remediation interventions (Glenthøj et al., 2020; Haugen et al., 2022; Myklebost et al., 2025; Ronold et al., 2023). However, studies find inconsistent rates of impairment on BRIEF-A in PD, and thus more investigations into the profile of srEF and its moderators are needed. One early study of BRIEF-A in schizophrenia found that patients had higher scores on Shift and Working Memory compared to healthy controls (Kumbhani et al., 2010). Informant-based reports indicated even higher scores, with all aspects of BRIEF-A showing significant differences compared to the informant-based reports of healthy controls, for a sample consisting of equal parts of both men and women with mean age in their forties.

Power et al. (2012) found that inpatients with “chronic schizophrenia”, with mean age in their mid-forties and approximately 1/3 women, showed clinically elevated scores (T-scores ≥ 65) on most measures of BRIEF-A when this was reported by a staff member in their institution, and that a global measure of srEF showed a small negative association to global functioning. In addition, Bulzacka et al. (2013) found that “stable” outpatients with schizophrenia, with mean age around 40 and 1/5 women, had significantly higher scores on all measures on the BRIEF-A compared to healthy controls, with the largest effects for the Initiate scale.

In more recent findings, Randers et al. (2020) reported medium to large differences on all BRIEF scores for both srEF and informant-based reports in 39 ultra-high risk for psychosis individuals with mean age in the early twenties with equal amounts of men and women, compared to controls, with the largest effect for difficulties with Working Memory. Glenthøj et al. (2020), in a similar but larger ultra high-risk group consisting of 132 individuals, found large differences on all aspects of BRIEF-A with largest deficits for the BRI. Haugen et al. (2021), reporting on three subscales in 66 people with schizophrenia, with mean age of 25 and 60 % males, noted that Working Memory was above clinically elevated levels (T-scores ≥ 65), while the Shift and Inhibit scales were not. Notably, this sample was recruited for a cognitive remediation study requiring srEF difficulties for inclusion (> T55 on the GEC). In a later investigation, including an additional 16 patients with “psychosis risk syndrome” Haugen et al. (2022) found clinically elevated T-scores on BRIEF-A scores for 81 participants, with all scales significantly different compared to normative tables. Only the Global Executive Composite (GEC) score of the BRIEF-A, and the Initiate and Working Memory scales were above the clinical cut-off values, however. A study looking at only the group with schizophrenia in this sample also reported GEC T-scores ≥65 (Øie et al., 2024). Similarly, Haugen et al. (2023) reported T-scores ≥65 on for Working Memory in the 73 participants completing the cognitive remediation intervention. In contrast, Van Aken et al. (2023) found average T-score for GEC to be approximately 58 in a large sample with a history of PD, mean age around 40, 1/3 women and 1/3 with diagnosed with schizophrenia, which is below clinical cut off. Thus, there seems to be support for srEF impairment in PD but differing results regarding subscales and degree of impairment. Given these heterogenous findings, more information about standardized scores on BRIEF-A in populations with PD and potential moderators and mediators for srEF impairments are needed.

There is some support that demographic characteristics could influence the heterogenous results displayed above. Although reporting standardized scores might control for the effects of education and gender, there was a tendency for females to report more cognitive difficulties in the mixed clinical sample in Groenman et al. (2022), but no other mediating or moderating factors emerged in their review. Haugen et al. (2023) did not find any effect of education on outcomes of cognitive remediation. A systematic review suggested that years of education are likely to protect against cognitive impairment in schizophrenia (Herrero et al., 2020), but how this relates to srEF difficulties is relatively unexplored. A systematic review found inconsistent associations between subjective and objective cognitive measurement and that higher cognitive ability could result in improved correspondence to self-rated cognitive function (Harris et al., 2023). Thus, gender, years of education and cognitive functioning could influence srEF in a PD population and account for some of the discrepant findings in the litterature.

The current investigation will add to the findings above through reporting norm-based scores for BRIEF-A in a large population of young Norwegian adults with PD and investigating how these are affected by available demographic and cognitive variables. Previous reports found clinically elevated T-scores on Initiate in a subset of this population (Raudeberg et al., 2023). Based on the findings above it is hypothesized that the current sample will show higher T-scores than the U.S. normative sample on all measures of BRIEF-A, and clinically elevated T-score on GEC and the Working memory Scale. In addition, the possible moderating roles of gender, and the effects of education and global cognition on BRIEF-A will be investigated.

## 2 Methods

The current study used anonymous archival data from a neuropsychological testing database of 462 patients referred for neuropsychological assessment from psychiatric hospitals in Bergen, Norway, from September 2013 to October 2018. The archival data set consists of The Repeatable Battery for the Assessment of Neuropsychological Status (RBANS) Index scores and subtest raw scores, BRIEF-A self-report T-scores, Norwegian Adult Reading Test (NART) and self-reported demographic variables including gender, age and education in years. The study is part of a research project evaluated and approved by the Regional Committee for Medical and Health Research Ethics (Reference Number: 2016/1330) and the Norwegian Data Protection Authority (Reference Number: 2017/575).

### 2.1 Participant characteristics

Inclusion criteria were minimum 18 years of age, Norwegian as first language, and a confirmed diagnosis of a schizophrenia spectrum disorder or undergoing a diagnostic evaluation due to manifest symptoms of schizophrenia, psychosis, or hallucinations. Diagnoses were according to the International Statistical Classification of Diseases and Related Health Problems, 10th revision (World Health Organization, 2016) and were decided by consensus of a team of psychiatrists and certified clinical psychologists. The most prevalent diagnoses were schizophrenia diagnosis (F20.0–F20.9) and schizoaffective disorder (F25.0–F25.9), accounting for about 50 % of the original sample of 462 patients. About 16 % of those had a diagnosis of psychotic disorder due to substance use (F1x.5). A minority (about 11 %) were awaiting diagnostic decision; whereas most would be diagnosed with a schizophrenia diagnosis (F20.0–F20.9). Patients with psychotic symptoms due to known affective disorders were excluded.

Due to patient confidentiality, and the anonymity of the dataset, individual data on diagnosis, symptom severity, medication use, admission duration, and treatment outcomes were not available. The sample consisted mainly of young individuals admitted to a secure inpatient specialized service for early-stage psychosis. Most were involuntarily admitted under mental health legislation. Admissions typically lasted around 12 weeks but ranged from a few weeks to over six months. Readmissions were common, often following medication discontinuation or relapse into substance use. Among substance-using patients, long-term polysubstance abuse and persistent psychotic symptoms were common. A detailed description of the archival sample can be found in Raudeberg et al. (2019). Given the purpose of the current study, inclusion criteria included completion of the BRIEF–A. Because not all assessments included the BRIEF-A, 260 of the 462 patients were eligible for participation (38.1 % females) after excluding protocols indicating invalid responding (*n* = 32). See Table 2 for patient characteristics.

### 2.2 Measures

BRIEF-A consists of a questionnaire comprised of 75 written statements that the respondent rates whether the specific behavior is never, sometimes, or often a problem. It takes about 10–15 min to administer and requires a reading level corresponding to fifth grade. Examples of statements are “I forget instructions”, and “I make careless mistakes” (Roth et al., 2005). The respondent's rating of each item (never, sometimes, or often) is given a score of one, two or three, respectively. The 75 items are divided into nine theoretically and empirically derived scales (Inhibit, Self-Monitor, Plan/Organize, Shift, Initiate, Task Monitor, Emotional Control, Working Memory, and Organization of Materials), two broader indices (Behavioral Regulation and Metacognition), and an overall summary score (Global Executive Composite). Table 1 provides which executive function(s) these scales and indices are intended to measure. BRIEF-A scores were interpreted by T-scores from the U.S. normative tables (Roth et al., 2005). T-scores ≥65 are 1.5 standard deviations above the normative mean and considered in the clinical range, scores of 60 are considered marginally impaired (Van Aken et al., 2022).

**Table 1:** Overview of BRIEF-A scales and indices and corresponding executive functions.

BRIEF-A scales and indices	Executive function and/or ability	
Inhibit	Measures the individual's ability to stop one's own behavior at the appropriate time (i.e., the ability to inhibit, resist, or not act on an impulse).	
Shift	Measures the ability to move freely from one situation, activity, or aspect of a problem to another, as the circumstances demand. It includes the ability to make transitions, problem-solve flexibly, switch or alternate attention, and change focus from one mindset or topic to another.	
Emotional Control	Addresses the manifestation of executive functions within the emotional realm and measures the ability to modulate emotional responses. Poor emotional control can be expressed as emotional lability or emotional explosiveness. Individuals with difficulties in this domain may have overblown emotional reactions to seemingly minor events.	
Self-Monitor	Measures a personal or social self-monitoring function or the extent to which one keeps track of his or her own behavior and its effect on others. Problems with monitoring are described in terms of failing to appreciate or have an awareness of one's own social behavior and the effect this might have on others.	
Initiate	Measures a person's ability to independently beginning a task or activity and generate ideas, responses, or problem-solving strategies. Poor initiation typically does not reflect noncompliance or disinterest in a specific task. Individuals typically want to succeed at a task, but they cannot get started. Individuals frequently report difficulties with getting started on tasks or chores, along with a need for extensive prompts or cues in order to begin a task or activity.	
Working Memory	Captures the capacity to actively hold information in mind for the purpose of completing a task or generating a response. Working memory is essential for a variety of everyday cognitive activities including carrying out multistep activities, implementing a sequence of actions, or following complex instructions. Individuals with weak working memory may have trouble remembering things (e.g., directions) even for a few minutes, lose track of what they are doing as they work, or forget what they are supposed to retrieve when instructed.	
Plan/Organize	Measures the ability to manage current and future-oriented task demands within the situational context. The Plan component of this scale relates to the ability to anticipate future events, implement instructions or goals, and develop appropriate steps ahead of time to carry out a task or activity. Planning often requires sequencing or stringing together a series of actions or responses. It is often described in terms of ability to start tasks in a timely fashion or to obtain, in advance, the correct tools or materials necessary to complete the activity. The Organize component of this scale relates to the ability to bring order to information, actions, or materials to achieve an objective. Individuals with organizational problems often approach tasks in a haphazard fashion or become easily overwhelmed by large amounts of information. They may have difficulty maintaining order in their environment or among their personal belongings.	
Task Monitor	Measures a problem-solving task-oriented, monitoring function. That is, the extent to which one keeps track of his or her own problem-solving success or failure. Problems with task-oriented monitoring are described in terms of failing to appreciate or have an awareness of one's own errors during such activities as problem-solving.	
Organization of Materials	Measures one's ability to maintain organization in his or her everyday environment, such as orderliness of work, living, or storage spaces such as desks, closets, and bedrooms. While this scale does not capture an executive function subdomain directly, the ability to keep one's environment organized is thought to reflect at least partly executive function abilities.	
	
Composite scales		
Behavior Regulation Index (BRI)	Summarizes the Inhibit, Shift, Emotional Control, and Self-Monitor scales. The BRI is interpreted as reflecting an individual's general ability to regulate or control his or her behavior and emotional responses, including appropriate inhibition of thoughts and actions, flexibility in shifting problem-solving set and adjusting to change, regulation of emotional responses, and, for adults and adolescents, monitoring of their own behavioral output.	
The Metacognition Index (MI)	Summarizes the Initiate, Working Memory, Plan/Organize, Task Monitor, and Organization of Materials scale. The MI can be interpreted as reflecting one's ability to get started on activity, to hold information in active working memory, to plan and organize problem-solving approaches, to complete tasks, and to maintain organization in the environment.	
Global Executive Composite (GEC).	A summary score of overall executive function across all scales.	
	
Validity scales		
Inconsistency	A measure of how many similar items are answered differently with differences in raw score > 6 indicating violation.	
Infrequency	A measure of how many items are missing from the report with ≥3 indicating violation.	
Negativity	Tendency for overly negative response pattern with ≥4 indicating violation.	

Note: Adapted from Roth, R. M., Isquith, P. K., & Gioia, G. A. (2014). Assessment of executive functioning using the behavior rating inventory of executive function (BRIEF). In *Handbook of Executive Functioning* (pp. 301–331).

**Table 2:** Descriptive statistics of age education, intelligence, and global cognition for the total sample and across gender.

	Total (*N* = 260)			Men (n = 161)			Women (n = 99)	
	M (SD)	Min–Max		M (SD)	Min–Max		M (SD)	Min–Max
Age	24.58 (5.79)	18–51		25.50 (5.42)	18–46		23.08 (6.08)	18–51
Education	12.59 (1.85)	9–18		12.74 (1.99)	9–18		12.34 (1.60)	9–17
Intelligence	104.85 (6.59)	91–119		106.01 (6.43)	93–119		102.53 (6.35)	91–115
Global Cognition	72.35 (18.66)	40–117		73.75 (17.74)	40–113		70.07 (19.94)	40–117

**Note.** Intelligence scores based on Norwegian Adult Reading Test for 60 women and 120 men. Global Cognition is based on The Repeatable Battery for the Assessment of Neuropsychological Status Total Scale Score.

**Table 3:** Descriptive statistics of BRIEF-A composite measures and clinical scales for the total sample and across gender.

	Total (*N* = 260)			Men (*n* = 161)			Women (*n* = 99)			Differences between men and women		
	M (SD)	Min–Max	% *T* ≥ 65	M (SD)	Min–Max	% T ≥ 65	M (SD)	Min–Max	% T ≥ 65	*t*	*p*	*d*
Composite scores												
Global Executive Composite (GEC)	60.92 (12.52)	35–88	39.6 %	58.73 (12.47)	35–88	32.3 %	64.47 (11.82)	37–88	51.5 %	3.73	<0.001	0.47
Behavioral Regulation Index (BRI)	57.10 (11.99)	35–89	27.7 %	54.89 (11.34)	35–82	19.3 %	60.70 (12.20)	37–89	41.4 %	3.82	<0.001	0.50
Metacognition Index (MI)	62.42 (12.42)	36–90	46.5 %	60.51 (12.56)	36–88	37.3 %	65.52 (11.60)	38–90	61.6 %	3.27	0.001	0.41

Clinical scales												

Inhibit	57.73 (12.01)	36–91	30.8 %	56.65 (11.52)	36–87	26.7 %	59.47 (12.64)	36–87	37.4 %	1.81	0.072	0.24
Shift	58.81 (12.32)	39–90	30.4 %	56.93 (12.47)	39–90	24.8 %	61.86 (11.48)	39–90	39.4 %	3.25	0.001	0.41
Emotional Control	54.04 (11.65)	38–93	20.8 %	51.45 (10.29)	38–93	12.4 %	58.25 (12.53)	38–92	34.3 %	4.54	<0.001	0.61
Self-Monitor	53.99 (11.74)	37–84	18.1 %	52.96 (11.20)	37–84	13.0 %	55.67 (12.44)	37–84	26.3 %	1.77	0.078	0.23
Initiate	64.17 (13.03)	37–89	48.1 %	62.44 (13.11)	37–89	41.6 %	66.98 (12.46)	37–89	58.6 %	2.80	0.006	0.35
Working Memory	63.28 (12.69)	39–89	50.8 %	60.63 (12.62)	39–89	42.2 %	67.59 (11.63)	39–89	64.6 %	4.53	<0.001	0.57
Plan/Organize	61.44 (12.12)	38–88	42.3 %	60.00 (12.49)	38–88	36.6 %	63.79 (11.15)	38–88	51.5 %	2.54	0.012	0.32
Task Monitor	59.12 (11.60)	36–93	30.0 %	57.69 (11.47)	36–93	23.6 %	61.44 (11.48)	36–93	40.4 %	2.56	0.011	0.33
Organization of Materials	54.95 (11.48)	36–81	19.2 %	53.69 (10.90)	36–81	13.7 %	57.00 (12.13)	36–81	28.3 %	2.22	0.028	0.29

**Note.** Means (M), standard deviations (SD), minimum and maximum scores, and percentage of participants with T-scores ≥65 are presented for the total sample and separately for men and women. Group differences were assessed using independent samples *t*-tests. Cohen's *d* indicates effect size. All scores are based on standardized T-scores from the Behavior Rating Inventory of Executive Function–Adult Version (BRIEF-A).

Intelligence (IQ) was estimated by the Norwegian version of the National Adult Reading Test (NART (Sundet and Vaskinn, 2008)). This test gives an estimate of premorbid IQ from participants correctly pronouncing several difficult words with high score equaling high performance. Estimated IQs were computed using regression-based norms published in Vaskinn et al. (2020).

The RBANS Total Scale scores were used as a measure of global cognition (Randolph, 2013). It is comprised of the sum of the immediate memory, visuospatial/constructional abilities, language, attention, and delayed memory indices, with high score indicates better performance.

### 2.3 Interpretive approaches and statistical procedures

IBM SPSS Statistics (version 29.0.2.0) were used for all analyses. Descriptive statistics and frequency tables were used to describe the sample, independent samples *t*-tests were used to test group differences for continuous variables and χ^2^ tests for categorial, and multivariate general linear models (GLM) was conducted for the BRIEF-A clinical scales, using gender, education, and global cognition as predictors. Age was excluded because the outcome (T-scores) is age-adjusted, thus age is inherently controlled for. Given that three predictors were tested across nine BRIEF-A clinical scales, Holm–Bonferroni corrections were applied to univariate follow-up analyses to control for Type I error. An α-value of *p ≤* .05 was used to determine statistical significance. Effect sizes were interpreted as small, medium, and large for Cohen's *d* at 0.2, 0.5, and 0.8 and for partial η^2^ at 0.01, 0.06, and 0.14, respectively. (Cohen, 1988)*.*

## 3 Results

A series of *t*-tests showed that men had more years of education (M = 12.74, *SD* = 1.99, *n* = 161) than women (M = 12.34, *SD* = 1.60, *n* = 99), *t*(240) = 1.76, *p* = .079, 95 % CI [−0.05, 0.84], Cohen's *d* = 0.21. Men also scored higher on NART IQ (M = 106.01, *SD* = 6.43) compared to women (M = 102.53, SD = 6.35), *t*(178) = 3.44, *p* < .001, 95 % CI [1.48, 5.48], Cohen's *d* = 0.54. It should be noted that only 60 women had NART IQ scores compared to 120 men, but a Levene's test indicated that the variances were not significantly different, *F*(1, 178) = 0.01, *p* = .913. There were no significant differences on the RBANS Total scale, *t*(258) = 1.55, *p* = .123, 95 % CI [−1.00, 8.35], Cohen's *d* = 0.20, with men (M = 73.75, *SD* = 17.74) and women (M = 70.07, SD = 19.94) scoring similarly.

In the entire sample 98 (37.7 %) participants used substances. More men (76, 47.2 %) used substances compared to women (22, 22.2 %), χ^2^(1) = 16.29, *p* < .001, φ = 0.25. Prevalence of substance use in men in the sample was comparable to prevalence in national patient registries (43.5 %) whereas prevalence for women was somewhat lower (22.2 % versus 30.3 %) (Nesvåg et al., 2015). Independent samples *t*-tests revealed statistically that substance users had fewer years of education (M = 12.03, *SD* = 1.84, *n* = 98) compared to non-users (M = 12.93, *SD* = 1.79, *n* = 162), *t*(258) = −3.87, *p* < .001, 95 % CI [−1.35, −0.44], Cohen's *d* = 0.50. There was also a significant difference between substance users (M = 60.82, *SD* = 12.64) and non-users (M = 55.86, *SD* = 11.52) for the Inhibit scale, *t*(258) = −3.29, *p* = .001, 95 % CI [−7.93, −1.99], Choen's *d* = 0.42. No other BRIEF-A scales or indices, nor estimates of intelligence and measures of global cognition differed significantly (*p*-values between 0.155 and 0.919).

### 3.1 BRIEF-A normative profile between males and females

The participants BRIEF-A T-scores are presented in Table 3. The entire sample does not reach clinical cutoff (i.e. *T* ≥ 65) for any scale or composite. Nevertheless, all values are higher than normative means and the Initiative and Working Memory scales are close to the clinical cutoff. Note that about half of the sample has a T-score ≥ 65 for the Metacognition Index (MI) and for the Initiate and Working Memory scales. Of the 260 participants, 86 (33.1 %) had none of the clinical scales ≥65, whereas 174 participants (66.9 %) had at least one scale ≥65 T-scores. A series independent samples of *t*-tests (see Table 3) revealed statistically significant differences between men and women across all BRIEF-A composite indices and seven out of nine clinical scales. Women consistently reported higher levels of executive functioning difficulties compared to men, particularly for the Working Memory and Emotional Control, and for the three composite scores (i.e., GEC, BRI, and MI).

### 3.2 Effects of demographic and cognitive variables on BRIEF-A scores

To examine the effects of gender, education, and global cognition on BRIEF-A scores, a multivariate General Linear Model (GLM) was conducted. Although preliminary analyses indicated that substance users differed from non-users on some variables, substance use was not included as a predictor in the model due to lack of statistical power in detecting possible gender and substance use interactions. Gender, education and global cognition (RBANS Total scale) had power estimates well above 0.80 for most outcomes, indicating that the GLM was sufficiently powered to evaluate the primary predictors of interest.

While all BRIEF-A scales showed significant deviations from normality (*W* = 0.95–0.98, all *ps* ≤ 0.008), skewness and kurtosis were within acceptable limits (skewness −0.49 to 0.24 and kurtosis −1.02 to −0.21). Thus, the deviations from normality were considered acceptable for GLM analysis given the sample size and the robustness of GLM to moderate normality violations. Linearity between predictors (i.e., gender, education, and RBANS Total Scale) and each dependent variable (i.e., the nine BRIEF-A clinical scales) was supported by scatterplots and low-order fit statistics (R^2^ = 0.03–0.10). Outliers were identified using Mahalanobis distance (99th percentile criterion: χ^2^ (9) = 26.22), identifying four outliers. These were inspected for punching errors and/or unusual BRIEF-A profiles but did not deviate from expectations and were thus retained. Box's M test was significant (*p* < .001); therefore, Pillai's Trace was reported for the multivariate effects.

The gender × RBANS Total interaction was significant at the multivariate level, Pillai's Trace = 0.066, *F*(9, 246) = 1.94, *p* = .048; all other multivariate effects were nonsignificant (*ps* ≥ 0.114). At the univariate level, significant effects emerged for gender on Initiate (*F*(1, 254) = 5.56, *p* = .019, partial *η*^*2*^ = 0.021), Education on Organization of Materials (*F*(1, 254) = 4.12, *p* = .043, partial *η*^*2*^ = 0.016), and RBANS Total on Shift (*F*(1, 254) = 3.92, *p* = .049, partial *η*^*2*^ = 0.015). The gender × RBANS Total interaction was significant for Self-Monitor (*F*(1, 254) = 7.73, *p* = .006, partial *η*^*2*^ = 0.030) and Initiate (*F*(1, 254) = 6.57, *p* = .011, partial *η*^*2*^ = 0.025); simple slope estimates indicated that higher RBANS Total was associated with lower Self-Monitor and Initiate scores in women, but not in men. After Holm–Bonferroni correction (*p* = .0056), none of the univariate effects remained significant.

In summary, although independent samples *t*-tests indicated significant gender differences on seven out of nine BRIEF-A clinical scales, these differences were not robust when examined in a multivariate GLM framework that controlled for educational attainment, and global cognition (RBANS Total scores). Gender and global cognition showed some significant multivariate interactions, but these effects were small, and none of the univariate associations for RBANS or its interactions with gender survived Holm–Bonferroni correction. The explanatory power of the model was modest, with R^2^ values ranging from 0.036 to 0.100 (≈4–10 % of variance explained).

## 4 Discussion

The current study found srEF difficulties to be in the marginally impaired range for a sample with PD. Not all measures were affected, and some specific aspects of Metacognition (Initiate, Working Memory, Plan/Organize) showed the largest normative deviations. None of the scales were above the clinical level for the whole group, in contrast to hypothesis. On average, females showed scores above clinical cutoff for Working Memory, Initiate and the Metacognition Index partially supporting the hypothesis. Although gender differences were found on some BRIEF-A scales, gender predicted BRIEF-A scores to a limited degree, with predictors not remaining statistically significant after controlling for the effects of educational attainment and global cognition, suggesting that other variables than gender could influence results.

In the U.S. standardization sample (Roth et al., 2005), higher educational achievement was associated with lower scores on the Emotional Control and Self-Monitor scales. Furthermore, women had higher scores on the Emotional Control scale compared to men, and conversely for the Initiate scale, where men had higher scores. Overall, education or gender accounted for less than 2 % of the variance in any scale, and the authors conclude that neither educational level nor gender should be considered a major factor in the interpretation of BRIEF-A scale scores. The result from the current study supports the overall conclusion that these factors are not major determinants of BRIEF-A scores.

### 4.1 BRIEF-A profile

On average, the entire sample had a BRIEF-A profile characterized of high T-scores on Initiate (T 64.2), Working Memory (T 63.3), Plan/Organize (T 61.4), and average scores on Self-Monitor (T 54.0), Emotional Control (T 54.0), and Organization of Materials (T 55.0). This pattern of high and average scores was similar for men and women and differed only in that women had somewhat higher scores. The BRIEF-A profile is consistent with the negative and cognitive symptoms seen in schizophrenia spectrum disorders. The most prevalent scales above clinical cutoff (T ≥ 65) were the Working Memory (50.8 %), Initiate (48.1 %), and Plan/Organize (42.3 %) scales, whereas the Self-Monitor (18.1 %), Emotional Control (20.8 %), Organization of Materials (19.2 %) scales had the lowest prevalences above cutoff. These results indicate that a large majority of the participants experience problems with remembering things, losing track of what they are doing, and have difficulties with getting started on tasks or chores; whereas the ability to regulate or control behavior and emotional responses seems less of a problem.

The Global Executive Composite showed a T-score just above the marginally impaired range. The Behavior Regulation Index showed < T-score of 60 as did all subscales on this measure. The current study indicates marginal self-reported deficits in Metacognition in early-stage psychosis. These deficits are of similar magnitude to those reported on objective tests of executive functioning in schizophrenia. However, norms could matter as suggested by a study by Løvstad et al. (2016), as healthy controls tended to have BRIEF-A scores 1/2–3/4 SD below the U.S. normative means, suggesting that scores of, for example, 56–64 might be in an actual clinical range and the authors underscores that a T-score of 65 should not be considered a necessary threshold to consider symptoms to be of clinical importance. However, Haugen et al. (2022) showed impairments well above clinical range in a similarly aged population, but this group was selected on account of srEF. In contrast, Van Aken et al. (2023) excluded reports with validity issues (elevated scores on negativety/inconsistency scales) that could have contributed the relatively low mean GEC score reported in their paper (T-score < 60), and had a mixed group PD with only 1/3 actually being diagnosed with schizophrenia. Therefore, different inclusion criteria and participant characteristics could likely influence the srEF profile in PD and could help explain the divergent findings in this field.

### 4.2 Gender

Females showed larger normative differences on BRIEF-A scores and scored above or approximating the clinical range for both GEC and MI. The BRI was also marginally impaired in this group, and it was the Inhibit and Shift scales that drove this effect. Females reported significantly higher srEF difficulties on all aspects of BRIEF scores except for Self-monitor and Initiate scales, with small to medium effects compared to males. Females did differ on some relevant aspects of cognitive function like education and estimates of intelligence (NART IQ), but not measures of global cognition (RBANS Total scale). Thus, perhaps premorbid IQ and eduction could influence srEF results (Herrero et al., 2020), but missing data makes this unexplored in the current study, and this notion also runs contrary to findings in previous studies that find a tendency of more premorbid impairments in men (Mendrek and Mancini-Marïe, 2016). NART is a limited IQ measure, however, and thus does not capture the full range of deficits experienced in the PD. More impairment in females has not been reported in the previous studies looking at BRIEF-A in PD and might account for heterogeneity in results and should be investigated by future studies. For instance, Glenthøj et al. (2020) and Randers et al. (2020) had a majority of female participants, while Van Aken et al. (2022) had over 65 % males, similar to the current study. This could support that gender is important for srEF deficits in PD and should be pursued in future investigations, however the limited availability of clinical variables in the current study makes it hard to rule out other confounding variables that could explain these differences.

### 4.3 Cognitive and demographical predictors of BRIEF-A scores

For women, higher scores on the RBANS Total scale (indicating better global cognition) were associated with lower scores on the Self-Monitor and Initiate scales of the BRIEF-A, whereas this was not found for men. Thus, global cognitive functioning was a better predictor of self-reported executive function for women than it was for men, at least for the Self-Monitor and Initiate scales. This has not been systematically reported in the PD literature and should be considered for future studies (Herrero et al., 2020). While some predictors were statistically significant, the models explained at most between 4 and 10 % of the variance, and much of the variability in executive functioning remains unexplained, which should be pursued by future research. Perhaps clinical variables like symptoms, illness duration, comorbidity, inpatient status and medication status could explain srEF, and these variables were not available for the current sample. The current study was also quite homogeneous with regards to age, and srEF likely will decrease with age for some patients (Power et al., 2012). Future studies should investigate self-reported executive function longitudinally to see how this aspect of cognitive function develops following psychosis onset, to identify groups in need of targeted treatments like cognitive remediation.

### 4.4 Strengths, limitations and future directions

This is the second largest study of srEF in PD known to the authors, and the first that investigates the effects of demographic variables like gender and education on BRIEF-A scores. Lack of clinical data like symptom measurements, inpatient status and other illness characteristics is a major limitation, and makes the findings considering gender hard to interpret reliably. Relationships between cognition and srEF could also be influenced by the fact that RBANS does not include measures of executive functions. Objective measures of executive functions should be included in future investigations of srEF and cognitive functioning. In addition, a more comprehensive measure of intelligence might have influenced results, since the NART is quite limited in only measuring verbal ability and might lack sensitivity to impairments seen in the PD group, and future studies might investigate associations between srEF and IQ with more robust IQ measures. Also, adding BRIEF-A Informant report would have potential to serve as a control and assessment of patients' awareness of their EF deficits. In addition, Norwegians have been found to have lower scores on BRIEF-A (Løvstad et al., 2016), and the U.S. norms might underestimate impairment in the current population (Raudeberg et al., 2019). Furthermore, the current study is of a relatively young population and cannot be generalized to older PD populations. Some might argue that srEF measures consisting of 75 items could be too demanding for PD populations with cognitive impairment and significant psychotic symptoms which could lead to inconsistent reporting. The current study excluded invalid protocols, which could be seen as a strength. None of the other cited studies using BRIEF-A mentioned these scales except for Van Aken et al. (2022), and thus not excluding invalid reports could have resulted in overreporting of srEF difficulties in some cited studies in this paper. Thus, excluding participant with validity issues in their protocols should be handled with care for the PD population. Finally, studies on srEF report different outcomes from similar or identical populations making the literature hard to summarize and navigate. Thus, reporting T-scores from all BRIEF-A scales is encouraged in future investigations. In conclusion, future studies should replicate findings in the current study, particularly for the effect of gender, and investigate srEF longitudinally to see how this develops following psychosis onset and identify groups in need of targeted treatments like cognitive remediation.

## 5 Conclusions

Patients with PD report, on average, difficulties in the marginally impaired range on BRIEF-A. These are largest for Metacognition Index and was clinically heightened for females. Future studies should replicate the current finding and look at additional moderators and mediators for subjective executive impairments like clinical variables. This could help identify individuals in need of personalized interventions like cognitive remediation.

## CRediT authorship contribution statement

**Eivind Haga Ronold:** Methodology, Investigation, Formal analysis, Conceptualization, Writing – review & editing, Writing – original draft. **Rune Raudeberg:** Project administration, Methodology, Formal analysis, Data curation, Conceptualization, Writing – review & editing, Writing – original draft.

## Declaration of Generative AI and AI-assisted technologies in the writing process

During the preparation of this work the authors used Microsoft Copilot to enhance language and readability. After using this tool, the authors reviewed and edited the content as needed and take full responsibility for the content of the published article.

## Declaration of competing interest

The authors declare that they have no known competing financial interests or personal relationships that could have appeared to influence the work reported in this paper.
